# Real-world experiences with obesity medication in the Netherlands: A mixed methods study among patients and professionals

**DOI:** 10.1016/j.obpill.2026.100319

**Published:** 2026-08-11

**Authors:** J.Th.C.M. (Anja) de Kruif, Mesut Savas, Marian A.E. de van der Schueren

**Affiliations:** a De Kruif Kwalitatief Onderzoek & Training, Niftrik, the Netherlands; b HAN University of Applied Sciences, Dept. of Nutrition, Dietetics and Lifestyle, Nijmegen, the Netherlands; c Erasmus MC, University Medical Center Rotterdam, Dept. of Internal Medicine, Division of Endocrinology, Rotterdam, the Netherlands; d Erasmus MC, University Medical Center Rotterdam, Hospital Pharmacy, Rotterdam, the Netherlands; e Wageningen University & Research, Division of Human Nutrition and Health, Wageningen, the Netherlands

**Keywords:** Health care professionals, Mixed methods, Obesity medication, Patients, Qualitative

## Abstract

**Background:**

Insight into how obesity medication (OM) is prescribed, accessed, and experienced in daily practice remains limited. This study explored prescribing practices, patient experiences and perceived barriers and facilitators regarding OM use in the Netherlands.

**Methods:**

This was an exploratory sequential mixed-methods study consisting of qualitative interviews followed by a cross-sectional questionnaire survey. Semi-structured interviews were performed with healthcare professionals and individuals using or considering OM (n = 14). Findings informed the development of a questionnaire completed by healthcare professionals (n = 102) and individuals using OM (n = 96).

**Results:**

The qualitative analysis generated five themes: (1) navigating access and reimbursement pathways, (2) negotiating professional roles and expertise, (3) balancing medication and lifestyle support, (4) living with OM: relief, stigma and legitimacy, and (5) uncertainty and future perspectives. Quantitative findings largely supported these themes. Overall, OM care was experienced as supportive and accessible. However, important challenges were identified, including: strict reimbursement criteria, a mandatory combined lifestyle intervention perceived as insufficiently tailored to individual needs, high out-of-pocket costs, uncertainty regarding tapering strategies, and differences between regular and private care pathways.

**Conclusion:**

OM is generally experienced positively within Dutch obesity care, but structural barriers remain. Improving access, reimbursement policies, professional education, and guidance regarding long-term treatment and tapering may contribute to more equitable and sustainable obesity care.

## Graphical abstract

**Figure:**
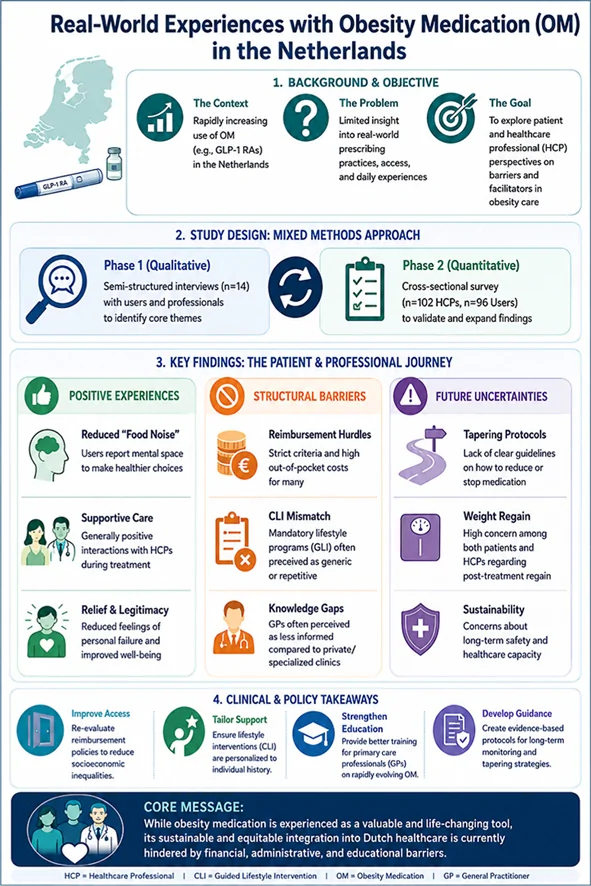

## 1 Introduction

Obesity is a major public health challenge worldwide and is associated with an increased risk of cardiovascular disease, type 2 diabetes mellitus, and other chronic conditions [1,2]. The prevalence of overweight and obesity has increased substantially over time. In the Netherlands approximately half of adults are living with overweight and one in six with obesity, while globally more than 1 billion people are living with obesity [2,3]. In recent years, pharmacological treatment options for obesity have gained increasing attention, particularly with the introduction of glucagon-like peptide-1 receptor agonists (GLP-1 RAs) and dual incretin therapies targeting both GLP-1 and glucose-dependent insulinotropic polypeptide (GIP) receptors. These medications have demonstrated substantial effects on weight reduction and metabolic outcomes through multiple mechanisms, including delayed gastric emptying, suppression of appetite, and reduction in food cravings and preoccupation with food [4,5]. Together with increasing awareness and availability of newer generation obesity medications (OM), these developments have contributed to a rapid rise in the number of users of these agents. The Dutch databank on healthcare statistics showed a 3.5 fold increase in numbers of individuals taking GLP-1 RA between 2020 and 2024 [6]. Importantly, these data include both diabetes and obesity-related indications. In 2025, the Dutch Pharmaceutical Key Figures Foundation recorded a total of 262,000 users of OM, a 57% increase compared to 2024. The number of patients using this medication for obesity rose by 88% to approximately 83,000 users [7]. Notably, these figures do not include over-the-counter use through online clinics or web shops.

In the Netherlands, guidelines provide recommendations for the prescription and use of OM in both primary [8] and specialist care [9]. These have been translated into practical interdisciplinary care pathways by the Partnership Overweight Netherlands [10], a national collaboration of healthcare professionals, health insurers, patient organisations, scientific organisations and public health partners.

In The Netherlands, the healthcare system is based on a managed competition model in which all residents are required to have health insurance and private health insurers compete under government-set rules [11]. Within this system, the Dutch National Health Care Institute (Zorginstituut Nederland) advises the government on medication reimbursement packages and conditions [12]. Reimbursement for OM is subject to strict eligibility criteria, including BMI thresholds, comorbidity requirements, and mandatory participation in a Combined Lifestyle Intervention programme (CLI), a programme lasting at least one year that focuses on promoting sustainable lifestyle changes through nutrition, physical activity, and behavioural support [13,14]. Patients must have participated in the CLI for at least one year without achieving sufficient weight loss and must meet the other eligibility criteria (BMI and co-morbidities) to entitle them to reimbursement for the medication. OM is also available through alternative care pathways, including online providers and private and commercial clinics, where the mandatory CLI is not required. However, these services are typically paid for out-of-pocket, and the level of clinical guidance and support may vary. Despite the rapidly increasing use of OM, insight into how OM is prescribed, accessed, and monitored in daily Dutch practice remains limited, both within and outside the regular healthcare system. The aim of this exploratory, mixed-methods study was therefore to explore how OM is used in daily practice in the Netherlands, including prescribing practices, patient experiences and perceived barriers and facilitators.

## 2 Methods

### 2.1 Study design

An exploratory sequential mixed methods design was used. First, qualitative interviews were conducted to explore experiences, roles, barriers, and facilitating factors related to OM use and guidance. Findings from this phase informed the development of a structured quantitative questionnaire distributed among healthcare professionals and users of OM.

### 2.2 Qualitative phase

#### 2.2.1 Participants and recruitment

Semi-structured interviews were conducted with stakeholders involved in prescribing, using, or guiding patients using OM. Purposive sampling was used to include different perspectives within the healthcare system and among patients (considering) using OM. Participants included healthcare professionals involved in obesity treatment, as well as individuals with experience using or considering OM. Healthcare professionals consisted of medical doctors, nurses, nurse specialists, and allied health professionals such as dietitians. Recruitment took place through professional networks, healthcare organisations, and social media platforms. All participants received written information and provided informed consent.

##### 2.2.1.1 Data collection

Semi-structured interviews explored experiences and perspectives regarding the use, prescription, and guidance of OM. Topics included access to OM, interactions with healthcare professionals, obesity care and reimbursement policies, lifestyle support, professional responsibilities, and future perspectives regarding OM within healthcare. The semi-structured format allowed participants to elaborate on issues they considered important and enabled exploration of new insights and themes. Interviews were conducted online or in person, audio-recorded, and transcribed verbatim.

#### 2.2.2 Data analysis

Data were analysed using reflexive thematic analysis [[15], [16], [17]]. Analysis involved iterative phases of familiarization, coding, theme development, review, definition and reporting. Themes identified in this phase informed questionnaire development. Reporting of the qualitative findings was informed by the values-based reporting approach proposed by Braun and Clarke [18].

#### 2.2.3 Reflexivity

Reflexivity was addressed through ongoing reflection on assumptions and prior knowledge throughout the analysis process. Credibility of the analysis was supported through systematic coding, repeated comparison with original transcripts, and the documentation of the analytic decisions.

### 2.3 Quantitative phase

#### 2.3.1 Questionnaire development

The questionnaire was developed based on the themes identified in the qualitative phase and included items on access to medication and reimbursement, prescribing practices, patient guidance and follow-up, multidisciplinary collaboration and barriers and facilitating factors. Standard survey design principles were applied [19,20]. The questionnaire included both closed-ended and open-ended questions. Although based on a single questionnaire framework, two tailored versions were developed using conditional routing and group-specific question blocks. The questionnaire was distributed among healthcare professionals and individuals using OM. Health care professionals included medical doctors, nurses/nurse specialists, and allied health professionals involved in OM care, recruited through professional networks, healthcare organisations, and social media. Individuals using OM were recruited through online platforms and patient communities. Participants were eligible if they were currently using OM or had experience with OM use. The questionnaire was administered online using QualtricsXM. The survey was open from December 8, 2025 to January 11, 2026.

#### 2.3.2 Data analysis

Responses were excluded if incomplete (<30%), duplicate or originated from outside the Netherlands. After data-cleaning, responses from 102 health care professionals and 96 individuals using OM were included. Due to conditional routing, the number of valid responses varied per question. Descriptive statistics were used to summarise data. Open-ended responses were categorised using thematic coding.

### 2.4 Integration

Integration occurred through a building approach, in which qualitative findings informed questionnaire development, and during interpretation, where qualitative and quantitative findings were compared at the thematic level.

## 3 Results

### 3.1 Qualitative findings

Fourteen participants were included in the qualitative phase: two individuals considering starting OM, five individuals with experience using OM, and seven healthcare professionals involved in obesity care (Table 1). All individuals using (or intending to use) OM interviewed were female. Thematic analysis identified five interrelated themes reflecting tensions and experiences surrounding OM care in the Netherlands [1]: navigating access and reimbursement pathways [2], negotiating professional roles and expertise [3], balancing medication and lifestyle support [4], living with OM: relief, stigma and legitimacy, and [5] uncertainty and future perspectives.

**Table 1:** Characteristics of participants using obesity medication in the qualitative interviews.

Medication user	Medication use and duration (weeks/months)	Sex	Age (years)	Educational level
1	No current user, considering OM	Female	Middle aged	Low/middle
2	No current user, considering OM	Female	Middle aged	Middle
3	Using OM for 5 weeks	Female	54	High
4	Using OM for 6 weeks	Female	57	Middle
5	Using OM for 3 months	Female	35	Middle
6	Using OM for 6 months	Female	Around 70	Middle
7	Using OM for 8 months	Female	37	Middle
**Health care professional**	**Background**	**Working area**		
1	Dietitian	Primary care	–	–
2	Dietitian	Primary care	–	–
3	Dietitian	Primary care	–	–
4	General practitioner	Primary care	–	–
5	Internal medicine specialist	Hospital care	–	–
6	Nurse practitioner	Private/commercial care	–	–
7	General practice assistant	Primary care	–	–

Abbreviation: OM, obesity medication.

#### 3.1.1 Navigating access and reimbursement pathways

Access to OM was frequently described as a complex and sometimes frustrating process shaped by reimbursement criteria, professional discretion, and financial resources. Across interviews, tensions emerged between formal policy requirements and participants’ lived experiences with obesity and previous attempts to manage their weight.

The mandatory combined lifestyle intervention (CLI) frequently functioned as a point of friction. While some participants valued peer support and guidance from a dietitian-lifestyle coach, most medication users experienced the programme as generic, insufficiently tailored to their personal situation and previous weight-loss attempts, repetitive, inflexible, or unnecessarily delaying access to medication.

Several participants expressed frustration at having to repeatedly demonstrate that they had already tried to lose weight before becoming eligible for medication: *“You're forcing me to do a year of CLI, even though you know what I've already tried.”* (P3)

One participant reported positive experiences with the dietician supervising the CLI programme, from whom she learned a lot and received continuous support. Although she initially felt reluctant to start, she eventually valued the program for helping her break habits and share experiences with others: “*Yes, I think CLI is always a good thing, just to share experiences with each other.”* (P7)

Healthcare professionals described reimbursement procedures as administratively burdensome and insufficiently aligned with clinical reality*: “The paperwork and reimbursement procedures often take more time than the actual patient care.”* (Nurse practitioner)

Professionals working in regular care referred to uncertainty regarding reimbursement procedures, prescribing criteria, and communication with insurance companies. In contrast professionals in private or commercial care described greater flexibility in prescribing OM, where treatment costs were generally fully covered by patients themselves.

Several participants explored private or commercial care pathways, which were often perceived as more accessible and responsive, particularly through digital communication and rapid follow-up. At the same time, both individuals using OM and professionals raised concerns regarding affordability and inequality, noting that out-of-pocket payment may limit access for individuals with fewer financial resources. Across interviews, access to OM was therefore not experienced solely as a medical issue, but also as shaped by organisational structures, reimbursement policies, and broader socioeconomic inequalities within obesity care.

#### 3.1.2 Negotiating professional roles and expertise

Participants described considerable variation in professional knowledge, confidence and responsibilities regarding OM. Across interviews, questions emerged regarding who should prescribe, supervise, and provide ongoing guidance for individuals using OM. Patients using OM frequently perceived general practitioners (GPs) as hesitant or insufficiently informed about rapidly evolving developments in obesity pharmacotherapy: *“GPs are behind. These developments are progressing so rapidly.”* (P3)

Healthcare professionals themselves described uncertainty regarding long-term treatment, tapering strategies, and the division of responsibilities between disciplines. Particularly in primary care, professionals described difficulties keeping pace with rapidly changing developments, increasing patient demand, and evolving reimbursement regulations. Several healthcare professionals also referred to uncertainty regarding long-term monitoring and the absence of clear tapering or discontinuation protocols. At the same time, allied health professionals emphasized that obesity should not be approached solely as an issue of medication prescription or individual responsibility. Dietitians in particular highlighted the importance of recognising obesity as a complex chronic condition requiring broader behavioural and psychosocial support; “*Medication can help, but if behaviour and underlying patterns remain unchanged, you are not addressing the root of the problem*.” (Dietitian).

Differences also emerged between regular and private care settings. Private providers were often perceived as more experienced in OM prescribing and follow-up care, while regular care was described as more constrained by reimbursement systems, limited consultation time, and guideline requirements. Across interviews, professional roles surrounding OM care appeared to remain in transition, particularly regarding long-term supervision, behavioural support, and interprofessional collaboration.

#### 3.1.3 Balancing medication and lifestyle support

Participants consistently described OM not as a standalone solution, but as part of a broader process of behavioural and lifestyle change. Many participants using OM reported long histories of dieting, coaching, or previous obesity treatments, often accompanied by frustration, shame, and repeated weight regain. OM was frequently described as creating “mental space” that enabled healthier choices and reduced constant preoccupation with food, often referred to as reduced “food noise”*: “When I'm bored, I open the fridge eighteen times and find out eighteen times that the content hasn't changed. That disappeared with the medication.*” (P4)

Several participants described that weight loss increased their motivation for physical activity and facilitated healthier eating patterns. At the same time, they emphasized that sustainable change still required behavioural adjustment and ongoing support. Healthcare professionals similarly stressed the importance of combining medication with lifestyle counselling, follow-up care, and behavioural guidance. Dietitians in particular expressed concern that expectations regarding medication effectiveness could overshadow the importance of long-term lifestyle support*: “People often think the medication itself is the solution, but sustainable change still requires guidance and support.”* (Dietitian)

At the same time, healthcare professionals also recognised that OM could support behavioural change by reducing constant hunger, emotional eating, and mental preoccupation with food, thereby creating opportunities for patients to engage more successfully in lifestyle interventions than they had previously been able to do.

Across interviews, tensions emerged between viewing OM primarily as a pharmacological intervention and understanding it as one component within broader obesity care. Participants frequently reflected on the need to balance medication use with sustainable behavioural change and long-term support.

#### 3.1.4 Living with OM: relief, stigma and legitimacy

Individuals using OM described obesity as a lifelong and emotionally demanding experience, often shaped by repeated attempts to lose weight, stigma, and feelings of personal failure. Across interviews, obesity emerged not only as a physical condition, but also as a socially and morally charged experience. Many participants described OM as reducing feelings of failure and loss of control around eating, accompanied by improvements in confidence, emotional wellbeing, and social functioning. Relief was frequently linked to reduced preoccupation with food and eating.

At the same time, participants described ongoing stigma surrounding both obesity and the use of OM. Some felt judged for using medication, particularly when OM was framed as an “easy solution” or as taking medication away from people with diabetes*: “If it was about willpower, I would have been slim long ago.”* (P5)

Several participants hesitated to disclose their medication use because they feared misunderstanding or negative judgement. Others emphasized that using OM did not diminish the effort required to achieve behavioural and lifestyle change.

Across interviews, obesity was frequently framed as remaining morally charged, both within healthcare encounters and broader societal discourse. Healthcare professionals similarly recognised that obesity continues to be insufficiently understood as a complex chronic condition*: “There is still this idea that obesity is simply a matter of personal responsibility, while in reality it is far more complex than that.”* (General practitioner)

Social media played a dual role in participants’ experiences. Some described online platforms as a source of recognition, support, and practical information, while others experienced social media discussions as stigmatising, polarising, or medically unreliable.

Questions of legitimacy also emerged regarding who should have access to OM and under what conditions. While participants generally viewed OM as a valuable treatment option, they simultaneously reflected on broader societal concerns regarding appropriate use, fairness, and responsibility within obesity care.

#### 3.1.5 Uncertainty and future perspectives

Participants expressed both optimism and uncertainty regarding the future role of OM within obesity care. Most individuals using OM described the benefits of treatment as outweighing concerns regarding side effects or potential long-term risks. Side effects were generally experienced as mild, manageable and acceptable in relation to improvements in weight, wellbeing, and quality of life.

Healthcare professionals, however, expressed greater uncertainty regarding long-term safety, prolonged use, tapering strategies and the risk of weight regain after discontinuation*: “We are only at the beginning of understanding what long-term obesity treatment with these medications will look like.”* (Internal medicine specialist)

Several professionals questioned whether existing healthcare systems are adequately organised for large-scale long-term OM treatment. Concerns included healthcare capacity, continuity of follow-up care, and the absence of clear long-term treatment frameworks.

Participants across groups emphasized the need for clearer guidelines, improved professional education, and more integrated obesity care pathways. Healthcare professionals particularly described uncertainty regarding responsibilities for prescribing, monitoring, tapering, and lifestyle support across disciplines and care settings.

Individuals using OM also reflected on future accessibility and affordability of treatment, expressing concern that financial barriers may increasingly determine who is able to continue treatment over time.

Across interviews, broader tensions emerged between prevention, lifestyle responsibility, chronic disease management, and medicalisation. While participants generally viewed OM as a valuable treatment option, they also reflected on questions regarding appropriate use, long-term dependency, and the future organisation of obesity care in the Netherlands.

### 3.2 Quantitative results

#### 3.2.1 Participant characteristics

The quantitative phase included 102 healthcare professionals and 96 participants using OM (Table 2). Healthcare professionals consisted of medical doctors, nurses, and allied health professionals, mainly dietitians. Participants using OM were predominantly female (94%), most were aged between 41 and 60 years, their present BMI (i.e., BMI at the time of completing the questionnaire, rather than their BMI before starting the medication) was median 31,3 (interquartile range 27,1–34,6) kg/m^2^ and the majority had used OM for less than one year.

**Table 2:** Characteristics of participants in the questionnaire.

Characteristic	Medical doctors (n = 21)	Nurses (n = 53)	Allied health professionals (n = 28)	Patients using obesity medication (n = 96)
Sex (female)	7 (33%)	53 (100%)	23 (82%)	90 (94%)
Age (years)				
21-30	–	–	–	7 (7%)
31-40	–	–	–	12 (13%)
41-50	–	–	–	30 (31%)
51-60	–	–	–	32 (33%)
>60	–	–	–	15 (16%)
Educational level				
High school/secondary vocational education	–	–	–	24 (25%)
Higher education	–	–	–	72 (75%)
Present BMI category (kg/m^2^)*				
<25	–	–	–	12 (13%)
25.0–29.9	–	–	–	24 (25%)
30.0–34.9	–	–	–	37 (39%)
35.0–39.9	–	–	–	12 (13%)
≥40	–	–	–	9 (9%)
Missing	–	–	–	2 (2%)
Professional background				
GP	10 (48%)	–	–	–
Medical specialist	5 (24%)	–	–	–
Elementary physician	5 (24%)	–	–	–
Researcher	1 (5%)	–	–	–
Dietitian	–	–	20 (71%)	–
Other AHP	–	–	6 (21%)	–
Other	–	–	2 (8%)	–
Work area				
Community care	10 (48%)	44 (83%)	16 (57%)	–
Hospital	5 (24%)	5 (9%)	5 (18%)	–
Private/commercial clinic	6 (29%)	2 (4%)	5 (18%)	–
Other	–	2 (4%)	2 (7%)	–
Duration of medication use				
0–3 months	–	–	–	22 (23%)
4–6 months	–	–	–	35 (37%)
7–12 months	–	–	–	31 (32%)
1–2 years	–	–	–	4 (4%)
3–4 years	–	–	–	4 (4%)
Medication type				
Tirzepatide				66 (69%)
Liraglutide				14 (15%)
Semaglutide				13 (14%)
Naltrexon-Bupropion				3 (3%)

Data are shown as numbers (percentage).

*BMI reflects BMI at the time of questionnaire completion and not BMI before initiation of OM.

Abbreviations: AHP, allied health professional; BMI, body mass index; GP, general practitioner.

##### 3.2.1.1 Access to medication and reimbursement pathways

Healthcare professionals in regular care generally reported adherence to national guidelines [8,9], whereas professionals working in private care or commercial clinics more frequently (57%) reported using alternative or no formal guidelines.

Only a minority of healthcare professionals (11%) had received education on OM during their initial training. Most medical doctors and nurses had completed additional OM-related training, which was generally perceived as improving confidence in prescribing and supervision. In contrast, 60% of allied health professionals had not attended any additional training.

**Fig. 1:**
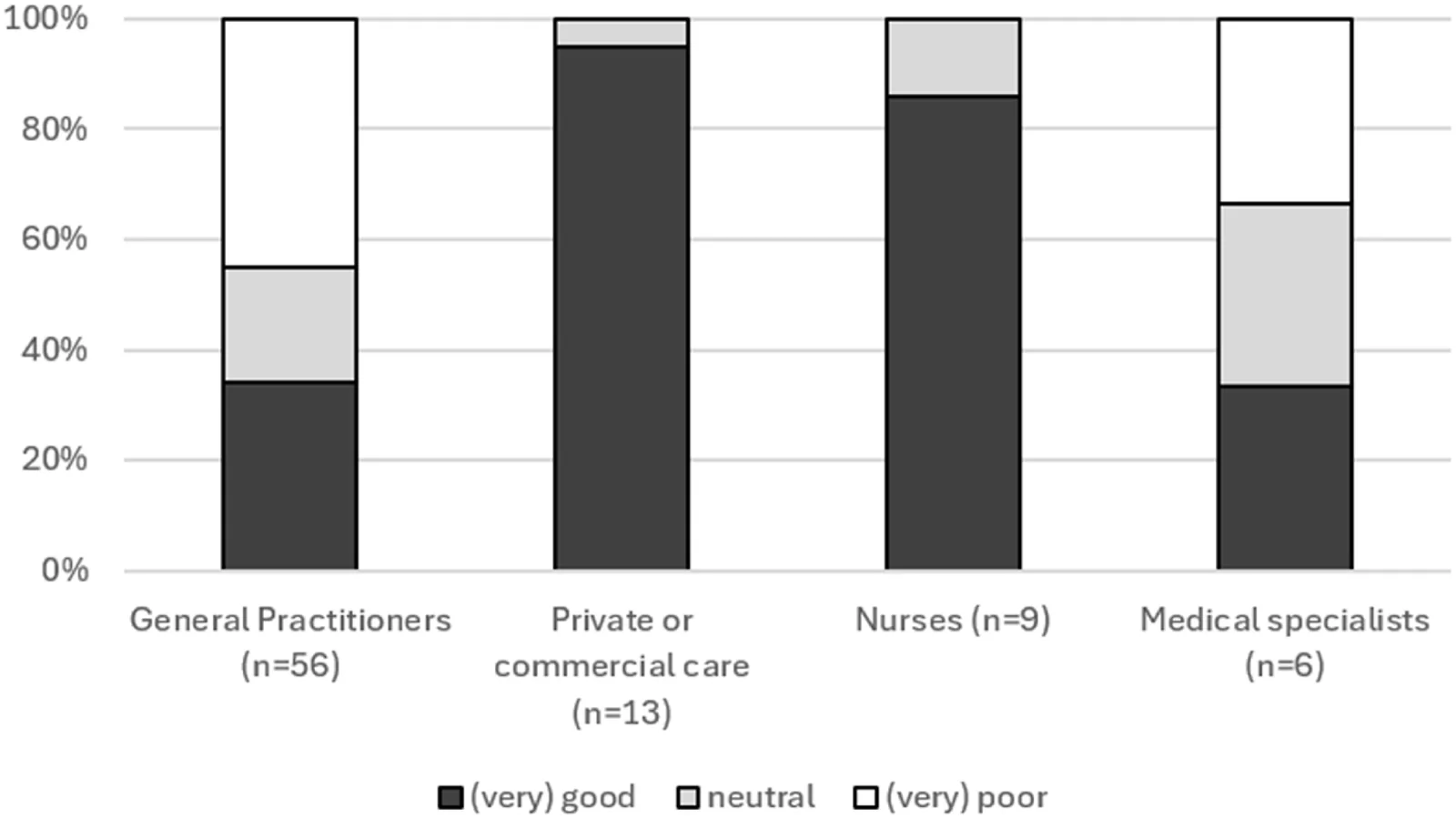
Rating of perceived knowledge of healthcare professionals by users of obesity medication (n = 82 respondents).

Ninety-six percent of individuals using OM reported having conducted their own research before starting the medication. Their perceived knowledge of healthcare professionals varied considerably and was rated highest for professionals in private or commercial care and lowest for GPs (Fig. 1).

Most individuals using OM (80%) reported feeling sufficiently involved in decisions regarding medication use and dosage. Most healthcare professionals were familiar with reimbursement eligibility criteria, including BMI thresholds and mandatory participation in a CLI. Only two nurses were unaware that only certain medication types were eligible for reimbursement. Administrative burden related to reimbursement procedures was frequently reported. In private and commercial care, reimbursement criteria were generally not applied, resulting in patients fully self-funding treatment. Many individuals using OM reported being highly familiar with reimbursement regulations and some felt they understood regulations better than their GP; only 15 of them (16%) were unaware of reimbursement conditions.

The majority of participants self-funded their treatment. These findings corresponded with the qualitative interviews, in which both individuals using OM and healthcare professionals described tensions surrounding reimbursement procedures, financial accessibility, and differences between regular and private care pathways.

##### 3.2.1.2 Professional roles, prescribing practices and eligibility criteria

Adherence to guideline recommendations regarding CLI participation varied across care settings. Among healthcare professionals in regular care, 47% of medical doctors and 13% of nurses reported prescribing OM without prior completion of a CLI programme, meaning that patients had to self-fund the medication. In private or commercial settings, CLI participation was generally not required.

Twenty-three individuals using OM (24%) had completed a CLI programme, five (5%) discontinued early, and 68 (71%) had never participated. Frequently mentioned reasons included already having sufficient knowledge regarding lifestyle change, lack of confidence in the programme, limited motivation, and not wanting to delay medication use for another year. Consequently, 80% of participants paid for OM themselves, often with noticeable impact on their daily budget. Healthcare professionals working in private or commercial care reported that their patients generally had a higher socio-economic status, whereas professionals in regular care described a more diverse patient population.

##### 3.2.1.3 Patient guidance, follow-up care and lifestyle support

Most healthcare professionals (73%) reported clear role division and agreements regarding OM-related patient care. Medical doctors and nurses were primarily involved in prescribing, dose adjustment, and monitoring side effects, whereas allied health professionals mainly focused on lifestyle counselling and behavioural support.

Consultation frequency was on average once every three months, although some healthcare professionals reported more limited or patient-initiated follow-up care. Participants who received guidance on dose adjustment (n = 44) rated this positively in 90% of cases, while support in managing side effects was rated as good or very good by 75% of participants who sought support (n = 24). Twenty-four participants using OM (25%) had been referred for additional lifestyle counselling, mainly to dietitians or lifestyle coaches. Tapering medication was identified as an important area of uncertainty. National protocols for tapering or discontinuation were lacking, resulting in healthcare professionals using individualised stepwise approaches. Only a minority of participants (n = 22, 23%) had discussed tapering or discontinuation with a healthcare professional. Among those who had, most perceived the explanation regarding tapering strategies as clear (76%). Some participants (7%) considered stopping OM because of side effects, high costs, or a wish to self-manage weight without medication. Both healthcare professionals and inidividuals using OM expressed concern regarding weight regain after discontinuation of OM. Similar uncertainties regarding tapering strategies, long-term follow-up, and weight regain emerged in the qualitative interviews.

##### 3.2.1.4 Barriers, facilitators and future challenges within the healthcare system

Healthcare professionals identified several barriers regarding OM care, particularly uncertainty about long-term effects and weight regain after discontinuation (70%), limited affordability for patients with financial constraints (58%), administrative burden (42%) and strict reimbursement criteria (36%). Professionals also referred to differences in information between patients and healthcare providers, sometimes leading to confusion or disagreement during consultations (28%).

Professionals working in private or commercial care more frequently expressed the view that current guidelines were too restrictive and limited access to treatment (90%).

Additional challenges emerging from open-ended responses included limited consultation time, incorrect medication use, unrealistic expectations due to media exposure, insufficient future healthcare capacity, overly strict CLI requirements, and the absence of integrated obesity care pathways. Facilitating factors included clear guidelines, good interprofessional collaboration, motivated patients, sufficient time for consultations, and sufficient access to information.

Individuals using OM similarly expressed concerns regarding accessibility and affordability of OM. Nineteen respondents (18%) expressed the wish for broader access to OM without mandatory prior CLI participation, while five (5%) criticized the high costs of private or commercial clinics. These findings corresponded with qualitative themes regarding accessibility, legitimacy of OM use, and structural inequalities within obesity care.

## 4 Discussion

This study explored how OM is used in daily practice in the Netherlands. The findings partially align with existing literature but also reveal several tensions within current obesity care. Similar to a previous qualitative study [21], individuals using OM reported structural barriers related to costs and reimbursement. These concerns were also acknowledged by healthcare professionals, particularly regarding patients who cannot afford to self-fund treatment.

Individuals using OM rated the professional knowledge of medical specialists and GPs relatively low, whereas professionals working in private/commercial clinics and nurses were generally rated more positively. For GPs and many medical specialists, obesity management represents only one aspect of a broad and diverse scope of clinical practice. Consequently, care for patients using OM constitutes a relatively smaller part of their daily work, unlike in private/commercial clinics where obesity management is a core focus of care. Given the rapid developments in obesity pharmacotherapy, keeping up to date may be more challenging for professionals for whom OM care is not part of routine practice. At the same time, medical doctors and nurses indicated that additional training improved their confidence in supporting patients using OM. In contrast, most allied health professionals reported having received little or no additional training in obesity medication treatment. This mismatch may represent an important gap in the delivery of evidence-based multidisciplinary care, particularly if prescribing clinicians assume that allied health professionals possess the necessary knowledge to adequately support patients using obesity medications. Future educational strategies should focus on equipping all members of the multidisciplinary team with the competencies required to deliver consistent, evidence-based obesity care.

Individuals using OM generally felt involved in decision-making and experienced health care professionals as supportive and accessible. Overall guidance during the medication trajectory was therefore experienced positively. This suggests that, within the Dutch healthcare context, professional support during OM use may be perceived more positively than reported in some other countries [22,23].

In the Netherlands, reimbursement for OM requires, among other criteria, participation in a CLI programme for at least one year without achieving sufficient weight loss. Although the CLI is intended to promote sustainable lifestyle change through nutrition, physical activity, and behavioural support, many participants experienced the programme as rigid and insufficiently tailored to individual needs and previous experiences with weight management. Previous Dutch research on the implementation of CLI in primary care also identified challenges, including limited awareness among GPs and uncertainty regarding its added value and long-term effectiveness [13]. Our findings extend these observations by showing that tensions surrounding the CLI are also experienced by individuals using OM, particularly when mandatory participation is perceived as being insufficiently aligned with previous weight-management experiences and as delaying access to medication. As a result, many participants in our study opted for self-funded treatment, often through private or commercial care pathways. Future research could compare treatment experiences and outcomes between individuals accessing OM through private care without mandatory prior CLI participation and those receiving OM after participation in a CLI.

Another possible explanation for choosing the private or commercial pathway may be that a substantial proportion of participants did not meet the BMI criteria for reimbursement when initiating OM treatment. Although baseline BMI was not assessed, approximately 40% of participants had a BMI below 30 kg/m^2^ at the time of questionnaire completion, and more than half had been using OM for less than 6 months. Together, these findings suggest that some participants may not have met the reimbursement criteria at treatment initiation; however, this remains an assumption that cannot be confirmed from our data.

Private or commercial clinics mainly attracted individuals with a higher socioeconomic status. Healthcare professionals working in regular care expressed concern regarding patients who may benefit from OM but are unable to access treatment because they do not meet reimbursement criteria or cannot afford the medication themselves. This financial barrier is particularly relevant given that the monthly costs of OM range from approximately €185 to more than €400 (depending on the medication and dosage) when treatment is not reimbursed [24].

This study also revealed considerable uncertainty surrounding tapering medication among both healthcare professionals and individuals using OM. The main concern was weight regain after discontinuation of medication. At present, standardised protocols for aftercare and tapering strategies are lacking, in part because it remains unclear whether OM can be discontinued at all [25].

A key strength of our research is its mixed-methods approach, integrating qualitative and quantitative data from both healthcare professionals and individuals using OM. This approach provided insight into how OM is accessed, prescribed, and experienced in daily Dutch practice. Future research should examine long-term OM use, tapering strategies, and experiences of groups that were underrepresented in the current study.

## 5 Limitations

Several limitations should be considered. Recruitment through online platforms and professional and patient networks likely attracted a selective audience. Our group of participants using OM mainly consisted of highly educated women, who self-funded their medication and therefore may not represent the broader population using OM in the Netherlands. Perspectives of individuals with lower socioeconomic status may therefore be underrepresented. Secondly, this study did not provide insight into OM obtained through illegal or unregulated channels. Health authorities warn that the safety of medication purchased outside regulated healthcare settings cannot be guaranteed [26,27]. Although only three respondents reported obtaining medication from a private seller, these numbers may underestimate the actual extent of unregulated OM use. In addition, the relatively small sample and unequal distribution across participant characteristics did not allow for meaningful subgroup analyses. Experiences related to medications obtained through unregulated channels were thus not comprehensively captured. Finally, we asked participants about their current weight; baseline body mass index prior to initiation of medication was unavailable. We were therefore unable to assess changes in BMI during treatment or the effectiveness of OM. Notably, thirteen percent of participants reported a current BMI below 25 kg/m^2^ while still using OM. Future research should investigate treatment trajectories among patients who achieve a healthy BMI while receiving OM, specifically examining how clinical decisions regarding continuation, dose adjustment, tapering, or discontinuation of treatment are made.

## 6 Conclusion

In conclusion, although OM care in the Netherlands was generally experienced as supportive and accessible, important structural and systemic challenges remain. These include strict reimbursement criteria, high out-of-pocket costs, considerable uncertainty regarding long-term treatment and tapering strategies, and a persistent mismatch between policy requirements and the needs and experiences of individuals seeking obesity treatment. Addressing financial barriers, improving clarity around reimbursement policies, and strengthening professional knowledge may contribute to more accessible and sustainable obesity care in the Netherlands.

**Clinical takeways**

• OM is generally experienced positively.
• Important structural barriers are identified, including strict reimbursement criteria, high out-of-pocket costs, and considerable uncertainty regarding long-term treatment and tapering strategies.
• Our findings provide actionable insights for clinicians, researchers, and policymakers working to improve the accessibility and sustainability of obesity care.

## Ethical approval and informed consent

The study was approved by the HAN Research Ethics Committee (ref ECO 716.11/25) and all participants received study information and provided informed consent prior to participation.

## Author contributions

Anja de Kruif and Marian de van der Schueren designed the study, developed the study material (interview guides, questionnaires), and obtained ethical approval. All authors recruited participants. Anja de Kruif performed and supervised the interviews. Marian de van der Schueren supervised the quantitative data analysis. All authors contributed to drafting the manuscript and approved its final version.

## Disclosures

Anja de Kruif: none.

Mesut Savas: none.

Marian de van der Schueren: none.

## Data sharing statement

The data from the qualitative study will not be made available for data sharing due to the sensitive nature of the information contained therein. Deidentified data from the quantitative study are available from the authors upon reasonable request.

## Declaration of artificial intelligence

ChatGPT (OpenAI) was used to improve grammar and language clarity and to create the graphical abstract. The authors reviewed and edited all AI-assisted outputs and take full responsibility for the content of the manuscript.

## Source of funding

Beyond payment to the research staff by the HAN University of Applied Sciences, De Kruif Kwalitatief Onderzoek & Training, Wageningen University & Research, Erasmus MC, University Medical Center Rotterdam, this research did not receive any specific grant from funding agencies in the public, commercial, or not-for-profit sectors.
